# Endoscopic submucosal dissection using a detachable snare for a large colorectal tumor with muscle retraction

**DOI:** 10.1055/a-2239-2913

**Published:** 2024-02-02

**Authors:** Shunsuke Horitani, Takeshi Yamashina, Natsuko Saito, Hironao Matsumoto, Masahiro Orino, Masataka Kano, Masaaki Shimatani

**Affiliations:** 1 50196 Division of Gastroenterology and Hepatology, The Third Department of Internal Medicine, Kansai Medical University Medical Center, Moriguchi, Japan

Large colorectal sessile tumors sometimes exhibit severe submucosal fibrosis with muscle retraction 1 . Endoscopic submucosal dissection (ESD) for tumors with muscle retraction tends to result in incomplete resection because submucosal dissection is difficult and carries a risk of perforation 1 . Conventional methods such as the double-tunnel and pocket-creation methods are useful for large sessile tumors with muscle retraction because good traction can be maintained and the dissection line is easier to recognize 2 3 . We report successful resection of a large colorectal tumor with muscle retraction at the center of the lesion using a detachable snare (PolyLoop Ligating Device; Olympus, Tokyo, Japan), which differs from conventional methods.

A 74-year-old man was referred to our hospital for the treatment of a large sigmoid sessile tumor, approximately 50 mm in diameter. ESD was performed using a DualKnife (KD-650U; Olympus). During submucosal dissection, extensive muscle retraction was observed in the center of the lesion ( Fig. 1 **a** ). To reveal muscle retraction without causing damage, mucosal incisions and submucosal dissection were repeated toward the distal side. Subsequently, the weight of the tumor was used to create appropriate traction on the retracted muscle by changing the patient’s position, followed by strangulation of the tumor with a detachable snare for extensive muscle retraction ( Fig. 1 **b** ). This method allowed good traction to be maintained and an appropriate dissection line to be identified, even in the presence of muscle retraction ( Video 1 ). The lesion was easily and completely resected en bloc without complications ( Fig. 2 ). The tumor measured 50×45 mm, and histological examination revealed a tubular adenocarcinoma in a tubulovillous adenoma, with free tumor margins.

**Fig. 1:**
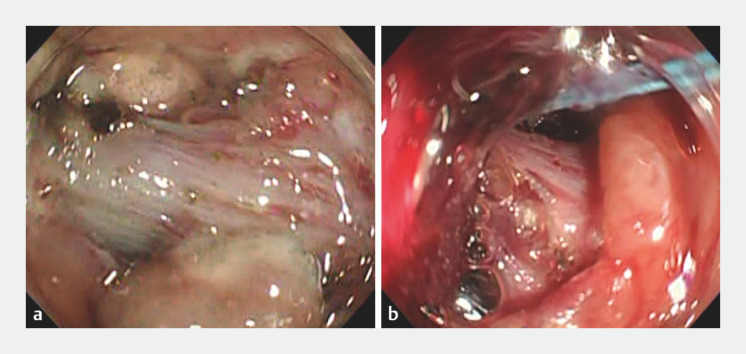
Resection of a large colorectal tumor with muscle retraction. **a** Extensive muscle retraction was observed in the center of the lesion. **b** Strangulation with a detachable snare for extensive muscle retraction provided good traction and facilitated identification of the dissection line.

Endoscopic submucosal dissection using a detachable snare for a large colorectal sessile tumor with muscle retraction. This method allowed good traction to be maintained and easy recognition of the muscularis for identification of a safe and appropriate dissection line.Video 1

**Fig. 2:**
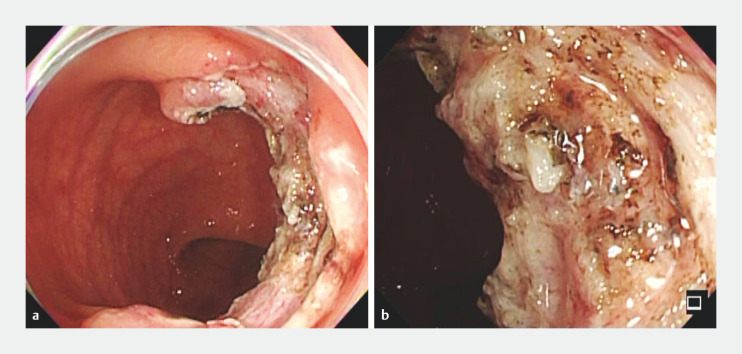
Endoscopy images. **a** After completion of submucosal dissection. **b** No complications occurred after resection of the retracted muscle.

ESD using the method of strangulation with a detachable snare for muscle retraction provides good traction and facilitates identification of the dissection line. This method is relatively easy and may reduce the treatment time compared with conventional methods.

Endoscopy_UCTN_Code_TTT_1AQ_2AD

## Notes

Endoscopy E-Videos https://eref.thieme.de/e-videos
*E-Videos* is an open access online section of the journal *Endoscopy* , reporting on interesting cases and new techniques in gastroenterological endoscopy. All papers include a high-quality video and are published with a Creative Commons CC-BY license. Endoscopy E-Videos qualify for HINARI discounts and waivers and eligibility is automatically checked during the submission process. We grant 100% waivers to articles whose corresponding authors are based in Group A countries and 50% waivers to those who are based in Group B countries as classified by Research4Life (see: https://www.research4life.org/access/eligibility/ ). This section has its own submission website at https://mc.manuscriptcentral.com/e-videos .
